# Practices in security and confidentiality of HIV/AIDS patients’ information: A national survey among staff at HIV outpatient clinics in Vietnam

**DOI:** 10.1371/journal.pone.0188160

**Published:** 2017-11-14

**Authors:** Nguyen Khac Hai, Saranath Lawpoolsri, Podjanee Jittamala, Phan Thi Thu Huong, Jaranit Kaewkungwal

**Affiliations:** 1 Hanoi Medical University, Hanoi, Vietnam; 2 Mahidol University, Department of Tropical Hygiene, Bangkok, Thailand; 3 Vietnam Authority of HIV/AIDS Control, Ministry of Health, Hanoi, Vietnam; Thai Red Cross AIDS Research Centre, THAILAND

## Abstract

**Introduction:**

Breach of confidentiality or invasion of privacy from the collection and use of medical records, particularly those of patients with HIV/AIDS or other diseases sensitive to stigmatization, should be prevented by all related stakeholders in healthcare settings. The main focus of this study was to assess practices regarding security and confidentiality of HIV-related information among staff at HIV outpatient clinics (HIV-OPCs) in Vietnam.

**Methods:**

A descriptive cross-sectional study was conducted at all 312 HIV-OPCs across the country using an online survey technique.

**Results:**

In general, the staff practices for securing and protecting patient information were at acceptable levels. Most staff had proper measures and practices for maintaining data security; however, the protection of patient confidentiality, particularly for data access, sharing, and transfer still required improvement. Most HIV-OPC staff had good or moderate knowledge and positive perceptions towards security and confidentiality issues. Staff who were not trained in the practice of security measures differed significantly from those who were trained (OR: 3.74; 95%CI: 1.44–9.67); staff needing improved knowledge levels differed significantly from those with good (OR: 5.20; 95%CI: 2.39–11.32) and moderate knowledge levels (OR: 5.10; 95%CI: 2.36–11.00); and staff needing improved perception levels differed significantly from those with good (i.e., with 100% proper practices) and moderate perception levels (OR: 5.67; 95%CI: 2.93–10.95). Staff who were not trained in the protection of data confidentiality differed significantly from those who were trained (OR: 2.18; 95%CI: 1.29–3.65).

**Conclusions:**

Training is an important factor to help raise the levels of proper practices regarding confidentiality and security, to improve knowledge and raise awareness about change among staff. The operation and management of HIV treatment and care in Vietnam are currently transitioning from separate healthcare clinics (HIV-OPC) into units integrated into general hospitals/healthcare facilities. The findings of this study highlight topics that could be used for improving management and operation of information system and revising guidelines and regulations on protection measures/strategies for data security and confidentiality of HIV/AIDS patients by Vietnam health authorities or other countries facing similar situations. Secure infrastructure and secure measures for data access and use are very important, worthwhile investments. The provision of continuous training and active enforcement and monitoring of the practices of healthcare personnel might lead to an improved understanding and acknowledegement of the importance of national policies/guidelines regarding HIV-related patient information.

## Introduction

Over 70% of people living with HIV (PLHIV) are concentrated in Sub-Saharan Africa and the Asia-Pacific [1, 2]. In Vietnam, HIV/AIDS is one of the 10 main causes of death [3]. In the last quarter of 2014, the Vietnamese government has committed to the HIV treatment target, the United Nations (UN) “90-90-90” program, which aims to stop the HIV pandemic by AD 2030. That means “*90% of people living with HIV will know their HIV status*, *90% of people who know their status are on HIV treatment*, *and 90% of all people on treatment will have undetectable levels of HIV in their body (known as viral suppression)*” [4]. Vietnam has made a concerted effort to increase the number of PLHIV to participate in antiretroviral therapy (ART) programs at local healthcare facilities, and the numbers have been increasing [5]. At the end of 2015, there were over 227,000 PLHIV in Vietnam, particularly those belonging to high-risk groups [6]. However, to join the ART program, patients must reveal their personal identifiable information—name, age, detailed address, national identification number, HIV status profile, etc. Several studies have identified associations between social stigma and the confidentiality/security of HIV/AIDS patient information [7–10]. Confidentiality is a concern for individuals seeking HIV-related healthcare services; when personal health information is not confidential or secure, HIV/AIDS patients are often reluctant to engage with these services [11–12].

The use of patient-identifiable data must strike a balance between maximizing the benefits of data usage and minimizing potential harm owing to inadvertent or malicious release of sensitive data [12]. Vietnam has adopted regulations and guidelines ensuring the security and confidentiality of HIV-related information; these include Decision No. 4159/QD-BYT, dated 13/10/2014, on the security of electronic health information at health facilities and the health industry [13], and the Health Ethics Regulation [14], among others.

Confidentiality and security measures are valuable because they reflect autonomy and control over personal information and free patients from the burdens of stigma, inequality, and discrimination [15]. Confidentiality concerns an individual’s right to protection of their data during storage, transfer, and use. Security is a collection of technical approaches that address issues covering physical, electronic, and procedural aspects of protecting any information that is collected [16]. Any breach of confidentiality or invasion of privacy should be prevented by all stakeholders. A study in Vietnam revealed that non-confidential healthcare processes can lead to serious consequences [17]. Although there have been numerous campaigns aimed at educating the public about HIV/AIDS, both globally and locally in Vietnam, evidence of the negative impacts of HIV-related stigma and discrimination are still reported [18].

In Vietnam, an HIV/AIDS care and ART program was established in 2000 by creating 312 HIV outpatient clinics (HIV-OPCs) nationwide. These clinics provide services exclusively for HIV patients, and patients at these clinics must provide personal identifiable-information to clinic staff. While Vietnam has escaped poverty and become increasingly prosperous, funding for HIV/AIDS programs has decreased rapidly with clearly reduced donor-sponsor support. This changing situation has obliged the Vietnam HIV/AIDS prevention system to be more streamlined [19]. The HIV-OPC system, which had been a separate HIV clinical setting, is currently in transition, and from 2017 will be gradually integrated into hospitals/general healthcare facilities. The change in management of the organization and delivery of ART and other HIV/AIDS services is a direct result of the reduction in donor funding. The operation and management resources for the HIV/AIDS program have to be increasingly subsidized by the Vietnamese government rather than the donors, and health insurance would be mandated to cover the cost of HIV patient treatment. At a hospital, HIV patients would be examined and treated like all other patients, while the distribution of antiretroviral (ARV) drugs would be implemented at the commune health station where patients live [19,20]. Personal health information will increasingly be shared with the health insurance agency when ARV drugs are administered and controlled under health insurance [5]. In addition, the data-sharing strategy has permitted health workers at the commune or hospital ward level to identify who is currently undergoing ARV treatment [21].

A balance is needed between the collection and sharing of individual-level data with the protection and safeguarding of such data, to optimize clinical care and to monitor and evaluate HIV services. The Joint United Nations Programme on HIV/AIDS (UNAIDS) investigated 98 mid- and low-income countries; reports varied substantially on issues of information governance and privacy, data storage and availability, data access, and data transfer [12, 22]. Several guidelines and standards on data security and confidentiality exist to facilitate the sharing and use of HIV surveillance data, covering access and roles, data sharing, physical and electronic data security, and security breaches [23–28]. No evidence-based information currently exists on how these concerns have been implemented in HIV-healthcare services in Vietnam. This study was conducted as a national survey, particularly among healthcare providers at HIV-OPCs, and focused on assessing healthcare staff practices regarding security/confidentiality of HIV-related information. In addition, staff personal characteristics, as well as their knowledge and perception levels regarding security measures and protection of data confidentiality, were assessed to determine their associations with practice activities.

## Materials and methods

### Population and samples

The target population for this study comprised staff working at HIV-OPCs. With respect to sample size, we required at least 384 participants for a confidence interval of 95%, with a 50% planned proportion estimate and absolute precision of 5%. The study was conducted at all 312 HIV-OPCs in Vietnam, with the expectation of about two staff members at each HIV-OPC participating in the study.

### Survey instrument

A descriptive cross-sectional study design was applied using an online survey, during March-June 2016. A structured questionnaire was used (S1 Appendix), with the content divided into four parts: Personal Information, Knowledge, Perception, and Practice. Items on the questionnaire were developed by adopting concepts, regulations of UNAIDS/PEPFAR (the United States President's Emergency Plan for AIDS Relief), and guidelines and regulations in Vietnam regarding confidentiality and security of HIV-related information, including policy and governance, data collection, data storage, data access and sharing, data transfer, and system security [12, 16, 23–30].

### Data collection and analysis

An e-mail containing a link to an online questionnaire was sent to the heads of the 312 HIV-OPCs, who then forwarded the link to their HIV-OPC staff. Staff members were asked to participate voluntarily in the study by completing the online form, which was automatically submitted to a database.

For the portion of the survey addressing knowledge (10 items), 1 point was given for each correct answer. Mean scores for knowledge were classified into three subgroups: Good (8–10 points), Moderate (6–7 points), and Needs Improvement (≤ 5 points). For the portion querying perception (9 five-point Likert scale items), a positive perception was established as responses of “Agree” and “Strongly Agree” with positive items, or “Disagree” and “Strongly Disagree” with negative items. Three subgroups for perception were also established: Good (8–9 points), Moderate (6–7 points), and Needs Improvement (≤ 5 points). To measure practice levels, the four-point Likert scale options were Always (≥ 80%), Often (≥ 50%), Rarely (< 50%), and Never (0%). Scoring of practice statements ranged from 1 to 4 for positive items and the reverse for negative items, respectively. The average scores were calculated for two sets of practices; i.e., security measures (4 items) and protection of data confidentiality (3 items). The average scores for the two types of practice were then classified into two subgroups representing staff with “proper practices” at an acceptable level (mean score ≤ 2) and “need improvement” practices (means score > 2).

Descriptive statistics were used to describe the characteristics, knowledge, perception, and practices of the respondents. Chi-square tests were used to compare the proportions between subgroups. Logistic regression was used to assess the strength of associations between respondent characteristics and their knowledge and perception levels with the levels of practices for security measures and protection of data confidentiality.

### Ethical considerations

The study was approved by the Vietnam Authority of HIV/AIDS Control, Ministry of Health of Vietnam, and the Ethics Committee of the Faculty of Tropical Medicine, Mahidol University, Thailand. For the first part on the online questionnaire, study participants who followed a link on the questionnaire were clearly informed of the study’s purpose, risks and benefits, and their voluntary involvement. Respondents were informed that they could stop at any time while completing the survey, that all data were kept confidential and secure and with no respondent identities, and that their answers to the survey would not affect their job.

## Results

### Respondent characteristics

From all 312 HIV-OPCs in 63 provinces, 400 staff from 238 (76%) in 56 (89%) provinces responded to the online survey (Table 1). Respondents included 178 (44.5%) doctors, 23 (5.8%) pharmacists, 154 (38.5%) nurses, and 45 (11.3%) administrative/other healthcare personnel. Among these professionals, 212 (53.0%) were female; higher female percentages were found among all professional categories, except doctors. Ages ranged from 21 to 60 years; age distributions varied across different professions. The distributions of time working in the HIV field and time working at the HIV-OPCs, also varied across professions. Overall, 296 (74.0%) of respondents had never been trained in data security and confidentiality. Based on their correct answers to the 10-items measuring knowledge of general principles and Vietnamese regulations on security and confidentiality of HIV data, the HIV-OPC staff were classified into three subgroups: 173 (43.2%) had good knowledge, 180 (45.0%) moderate knowledge, and 47 (11.8%) needed improvement. Although there was no statistically significant difference between the different professions, higher percentages of good knowledge were found among doctors and administrative/other professionals. Similarly, based on their ratings for the 9 items measuring perceptions regarding perceived benefits and threats to protect data security and confidentiality of HIV data, the HIV-OPC staff were classified into three subgroups: 104 (26.0%) had good perception, 185 (46.2%) moderate perception, and 111 (27.8%) needed improvement. Again, no statistically significant difference was evident between professions, but a higher percentage of good perception level was found among administrative/other professionals.

**Table 1:** Characteristics of survey respondents (N = 400).

Characteristics		Total (N = 400)	Professions								p-value
			Doctor (N = 178)		Pharmacist (N = 23)		Nurse (N = 154)		Admin & Other (N = 45)		
			n	%	N	%	n	%	n	%	
Sex	Male	188	110	61.8%	6	26.1%	53	34.4%	19	42.2%	<0.01
	Female	212	68	38.2%	17	73.9%	101	65.6%	26	57.8%	
Age group (Years)	20–30	132	21	11.8%	12	52.2%	72	46.8%	27	60.0%	<0.01
	31–40	131	53	29.8%	7	30.4%	61	39.6%	10	22.2%	
	41–50	83	65	36.5%	2	8.7%	10	6.5%	6	13.3%	
	51–60	54	39	21.9%	2	8.7%	11	7.1%	2	4.5%	
Time working in HIV field (Years) (N = 395)	1–5	228	85	47.8%	15	65.2%	94	61.8%	34	81.0%	<0.01
	6–10	130	68	38.2%	8	34.8%	50	32.9%	4	9.5%	
	> = 11	37	25	14.0%	0	0.0%	8	5.3%	4	9.5%	
Time working in the OPC (Years) (N = 391)	1–5	255	106	59.9%	15	65.2%	102	67.1%	32	82.1%	0.09
	6–10	119	60	33.9%	8	34.8%	46	30.3%	5	12.8%	
	> = 11	17	11	6.2%	0	0.0%	4	2.6%	2	5.1%	
Training	Yes	104	50	28.1%	6	26.1%	36	23.4%	12	26.7%	0.81
	No	296	128	71.9%	17	73.9%	118	76.6%	33	73.3%	
Knowledge level	Good	173	79	44.4%	8	34.8%	61	39.6%	25	55.6%	0.39
	Moderate	180	75	42.1%	12	52.2%	75	48.7%	18	40.0%	
	Need improvement	47	24	13.5%	3	13.0%	18	11.7%	2	4.4%	
Perception level	Good	104	45	25.3%	3	13.0%	39	25.3%	17	37.8%	0.36
	Moderate	185	83	46.6%	11	47.8%	75	48.7%	16	35.5%	
	Need improvement	111	50	28.1%	9	39.1%	40	26.0%	12	26.7%	

### Practices for protection and safeguarding of confidential PLHIV information

Regarding security measures (Table 2), about 90% of HIV-OPC staff always kept patients’ paper records in a secured location. For entering patient data on personal computers, about 75% did not do so, but another 8% always or often did it while 17% rarely did so. About 53% of HIV-OPC staff followed the policy of periodically changing password while another half rarely or never did so. For practices of safeguarding the system, 40% of staff always and 42% often installed antivirus-software on computers containing HIV data.

**Table 2:** Proportions of security and confidentiality practices.

Respondent’s Practices	Always (>80%)		Often (>50%)		Rarely (< = 50%)		Never (0%)	
	n	%	n	%	n	%	n	%
**Security Measures**								
*Keeps patient’s paper records in a secure*, *locked cabinet or room at the end of each day*	362	90.4%	36	9.0%	1	0.3%	1	0.3%
*Enters patient data on a personal computer*	11	2.8%	20	5.0%	70	17.4%	299	74.8%
*Periodically sets and changes password for computers where patient information is stored*	56	14.0%	155	38.8%	143	35.8%	46	11.4%
*Installs and uses antivirus software on computers containing sensitive data of HIV patients*	160	40.0%	168	42.0%	41	10.3%	31	7.7%
**Confidentiality Protection**								
*Shares patient information with other trusted healthcare personnel for consultation purposes*	3	0.7%	29	7.3%	137	34.3%	231	57.7%
*Sends reports with HIV patient information via unsecured networks (not authenticated)*	4	1.0%	50	12.5%	92	23.0%	254	63.5%
*Obtains consent from HIV patients to share their personal information (e*.*g*., *with a relative or close friend)*	82	20.5%	74	18.5%	60	15.0%	184	46.0%

The practices on protection of confidential data of the HIV patients were not fully compliant with the national policy and regulation (Table 2). For data sharing and transfer, 64% of respondents never sent reports containing HIV-related information via unauthenticated networks whereas 14% often or always did so. About 58% of respondents never shared patient information with other trusted healthcare personnel for consultation purposes; however, 8% often or always did so. Comparing across professional categories (table not shown), there were differences among professionals in this area: 70% of pharmacists never shared such information compared with 56%–60% of nurses and doctors and 47% of other staff; 7% of doctors and 13% of nurses often or always did so. In practice, up to 46% of respondents never obtained consent from the patient to share their personal information with others, whereas about 39% always or often did so. No significant differences were reported among professionals (table not shown).

### Associations of staff characteristics with security and confidentiality practices

The associations of staff characteristics with their practices on security measures and protection of patient data confidentiality were analysed separately (Tables 3 and 4). From the average scores of their practice rating on 4-items regarding security measures, the HIV-OPC staff were classified into two subgroups: 348 (87%) had proper practice and 52 (13%) needs improvement in their current practices (Table 3). Male and female staff were not statistically significant in their practices. Similarly, there were no statistically significant differences among professionals, though lower percentage of pharmacists who had proper practice scores was observed. Time working in HIV field and time working at OPC were not significantly associated with proper practices on security measures; however, percentages of proper practices appeared to increase with longer working time. Staff who were not trained on security were significantly different from those who were trained (OR: 3.74; 95%CI: 1.44–9.67). Staff with needs improvement knowledge level were significantly different from those with good knowledge level (OR: 5.20; 95%CI: 2.39–11.32) and moderate knowledge level (OR: 5.10; 95%CI: 2.36–11.00). Similarly, staff with needs improvement perception level were significantly different from those with good perception level (with 100% proper practices) and moderate perception level (OR: 5.67; 95%CI: 2.93–10.95).

**Table 3:** Associations of practices on security measures, according to different staff characteristics.

Characteristics		Practices on Security Measures				Odds Ratio (95% CI)
		Need improvement (N = 52)		Proper practice (N = 348)		
		n	%	n	%	
Sex	Male	18	9.6%	170	90.4%	**1.80 (0.98–3.32)**
	Female	34	16.0%	178	84.0%	**1**
Professions	Doctor	24	13.5%	154	86.5%	**0.80 (0.29–2.23)**
	Pharmacist	5	21.7%	18	78.3%	**0.45 (0.12–1.75)**
	Nurse	18	11.7%	136	88.3%	**0.94 (0.33–2.70)**
	Admin & Other	5	11.1%	40	88.9%	**1**
Age group (Years)	20–30	19	14.4%	113	85.6%	**1.03 (0.42–2.53)**
	31–40	20	15.3%	111	84.7%	**0.96 (0.40–2.35)**
	41–50	5	6.0%	78	94.0%	**2.71 (0.84–8.79)**
	51–60	8	14.8%	46	85.2%	**1**
Time working in HIV field (Years)	1–5	36	15.8%	192	84.2%	**0.31 (0.07–1.32)**
	6–10	14	10.8%	116	89.2%	**0.47 (0.10–2.18)**
	> = 11	2	5.4%	35	94.6%	**1**
Time working in the OPC (Years)	1–5	36	14.1%	219	85.9%	**0.81 (0.18–3.70)**
	6–10	14	11.8%	105	88.2%	**1.00 (0.21–4.84)**
	> = 11	2	11.8%	15	88.2%	**1**
Training	Yes	5	4.8%	99	95.2%	3.74 (1.44–9.67)*
	No	47	15.9%	249	84.1%	**1**
Knowledge level	Good	17	9.8%	156	90.2%	5.20 (2.39–11.32)*
	Moderate	18	10.0%	162	90.0%	5.10 (2.36–11.00)*
	Need improvement	17	36.2%	30	63.8%	**1**
Perception level	Good	0	0.0%	104	100.0%	-
	Moderate	15	8.1%	170	91.9%	5.67 (2.93–10.95)*
	Need improvement	37	33.3%	74	66.7%	**1**

* p-value < 0.05.

**Table 4:** Associations of practices on protection of confidentiality, according to different staff characteristics.

Characteristics		Practices on Confidentiality				Odds Ratio (95% CI)
		Need improvement (N = 136)		Proper practice (N = 264)		
		n	%	n	%	
Sex	Male	65	34.6%	123	65.4%	**0.95 (0.63–1.44)**
	Female	71	33.5%	141	66.5%	**1**
Professions	Doctor	60	33.7%	118	66.3%	**1.19 (0.61–2.35)**
	Pharmacist	7	30.4%	16	69.6%	**1.39 (0.47–4.06)**
	Nurse	52	33.8%	102	66.2%	**1.19 (0.60–2.37)**
	Admin & Other	17	37.8%	28	62.2%	**1**
Age group (Years)	20–30	43	32.6%	89	67.4%	**0.59 (0.28–1.24)**
	31–40	48	36.6%	83	63.4%	**0.49 (0.24–1.03)**
	41–50	33	39.8%	50	60.2%	0.43 (0.20–0.94)*
	51–60	12	22.2%	42	77.8%	**1**
Time working in HIV field (Years)	1–5	73	32.0%	155	68.0%	**0.90 (0.42–1.92)**
	6–10	51	39.2%	79	60.8%	**0.66 (0.30–1.44)**
	> = 11	11	29.7%	26	70.3%	**1**
Time working in the OPC (Years)	1–5	85	33.3%	170	66.7%	**0.27 (0.06–1.19)**
	6–10	47	39.5%	72	60.5%	0.20 (0.04–0.93)*
	> = 11	2	11.8%	15	88.2%	**1**
Training	Yes	23	22.1%	81	77.9%	2.18 (1.29–3.65)*
	No	113	38.2%	183	61.8%	**1**
Knowledge level	Good	63	36.4%	110	63.6%	**1.08 (0.56–2.11)**
	Moderate	55	30.6%	125	69.4%	**1.41 (0.72–2.75)**
	Need improvement	18	38.3%	29	61.7%	**1**
Perception level	Good	35	33.7%	69	66.3%	**1.40 (0.80–2.43)**
	Moderate	55	29.7%	130	70.3%	1.67 (1.02–2.74)*
	Need improvement	46	41.4%	65	58.6%	**1**

* p-value < 0.05.

From the average scores of their practice rating on 3-items regarding protection of patient data confidentiality, the HIV-OPC staff were classified into two subgroups: 264 (66%) had proper practice and 136 (34%) needs improvement in their practices (Table 4). Again, the staff practices regarding protection of data confidentiality were not significantly associated with sex, professional categories, time working in HIV field and at OPC, Staff who were not trained on such issues were significantly different from those who were trained (OR: 2.18; 95%CI: 1.29–3.65). Regarding the protection of data confidentiality, knowledge and perception levels were not significantly associated with such practices, even though the percentages of staff with proper practices were lower among those with needs improvement than the ones with moderate and good knowledge or perception levels.

## Discussion

The results of this study suggested that security and confidentiality practices among HIV-OPC staff potentially increase the risk of revealing patient information. There has been a question whether separate HIV clinical settings, as compared with general hospital settings, would result in a greater or lower risk of breaching confidentiality. With the new approach when the treatment of ARV is conducted at a public health facility and ARV drugs are administered by a commune health station, HIV/AIDS patients are exposed to several different levels of healthcare personnel and other people, including non-HIV-infected patients. Therefore, the risk of exposing confidential information is higher, and ensuring security and confidentiality is more difficult. Before the changes in policy and management in 2017, all HIV-OPCs operated as separate units; the HIV information management software was a specific system and different from the information management system used at the public hospital and general healthcare facilities. From 2017 onwards, when the integration of HIV-OPCs into public hospital settings is implemented, a good management information system is essential to ensure the security and confidentiality of HIV/AIDS patients data that become part of the large information systems normally used in general hospitals. In the literature, an exit survey demonstrated that some integrated sites had raised concerns about patient confidentiality [31]. A study of awareness, experiences, and attitudes among staff in clinical settings suggested different environments might lead to leaks of confidential information, including the lack of private rooms for patient examination and the use of public-access computerized systems [32]. Even where a system is physically secure, multidisciplinary staff not involved in HIV/AIDS patient care may have access to detailed patient information [15, 32]. However, it can be concluded that any setting has a potential for violating patient confidentiality, if appropriate protective measures are not in place.

The majority of HIV-OPC staff had good or moderate levels of knowledge (88%) and positive perceptions (72%) regarding issues about security measures and disclosing/ensuring patient confidentiality. It should be noted that their knowledge and perception levels on such issues did not differ across profession categories, which may have different roles and responsibilities for accessing and using patient information. The professionals also did not differ in terms of training in security and confidentiality issues; however, only about a quarter of all professionals had been trained.

As a general rule in data security measures, personal information should be collected, stored, and disseminated in accordance with applicable national regulations [33]. Several guidelines for HIV programs have suggested planning and management for both physical and data security [13, 23–30, 33, 34]. One study reported that 80% of AIDS surveillance offices in the United States have a security guard during non-working hours and 44% have fireproof cabinets [35]. Vietnam has similar regulations and guidelines on physical security for HIV/AIDS surveillance [13, 34, 36, 37]. As a simple physical security measure in this study, nearly all staff (90%) were well aware of and practiced keeping patients’ paper records in secured locations, which requires no complex information technology or skill. On the contrary, about 25% of HIV-OPC staff had entered patient data on personal computers; the practices for electronic data security that require computer applications/and/or electronic services had raised a risk management concern, since only 14% of staff rigorously changed passwords periodically and about 40% always installed and used antivirus software on computers containing patient data. These policies should be emphasized and enforced. Implementation of other manageable data security measures should be considered, as in another study showing that about 74% of HIV/AIDS offices in the United States installed security locks and 54% installed specialized software to control computer access [35].

Regarding protection of confidentiality of patient information in practice, about 39% of HIV-OPC staff always or often obtained patient consent before sharing personal information. However, for staff who reported never obtaining consent, this might be partially because these personnel had no role in using such information. In other studies in Vietnam [38, 39], 38% of PLHIVs reported that their HIV status had been disclosed without their consent. This incidence, varying from 15%-50%, was also reported in other Asian and African countries [40–42]. Interestingly, a study among medical students reported that about three-fourths believed they had the right to inform the sexual partner of an HIV-positive patient of the patient’s HIV status [43]; however, another recent study reported that 70% of community members recommended maintaining patient confidentiality [44]. Confidentiality of patient information is based on trust between healthcare providers and their patients [15]. The general principle justifying the reasons for access and disclosure of minimum patient-identifiable information should be that it is acceptable only when absolutely necessary and on a strict need-to-know basis [12, 15, 22]. About 58% of HIV-OPC staff never shared patient information with other trusted healthcare personnel for consultation purposes, but some doctors and nurses often or always did so. This reflects a common practice among doctors and nurses, who have direct access to patient records for providing treatment and care, thus raising the potential for breaching patient confidentiality. The administrative staff who perform patient registration and pharmacists who request certain information about a patient’s condition might sometimes disclose patient information. A study among homo/bisexual men indicated that disclosure of their HIV status was used as a mechanism for coping with the disease [45]. Similarly, a study among clinical staff stated that they felt justified in disclosing information to other health personnel on a need-to-know basis [32]. A study among HIV/AIDS patients and other stakeholders in healthcare services suggested that the acceptability of data sharing depends on trust in how the data will be used [46]. As suggested in all guidelines, there should be appropriate technical and organizational measures in place to prevent unauthorized use and transfer of data to unrelated settings [23–27]. About one-third of HIV-OPC staff had shared/transferred patient information via unsecured mechanisms. It is critical to consider the protection of patient information as the responsibility of healthcare providers [47]; therefore, more robust methods should be implemented, including encryption and strong authentication mechanisms [23, 26].

In examining the associations of HIV-OPC staff characteristics with their security and confidentiality practices, differences were observed among those who had been trained and those who had not. Those who had been trained on the concepts of data security and safeguarding patient data by the Vietnam Ministry of Health had better practices for both security measures and protection of personal health information. A systematic literature review reported that the implementation of legal requirements in dealing with security and confidentiality of sensitive data was mostly due to non-technical measures, including education/training program and raising awareness of such issues [48]. In another systematic review study identifying the needs of healthcare professionals to receive effective training in patients’ information confidentiality, it was suggested that health authorities must address the needs and provide training on the policies pertaining to confidentiality while the healthcare personnel need to have the proper skill set to ensure the security and confidentiality of patient health records [49]. The best solution for compliance with policies regarding security and confidentiality was to offer healthcare professionals educational tools, whereby healthcare organizations would provide regular updates to all professional health staff regarding the policies and procedures to prevent the exposure of sensitive data [48]. There were some studies reported the associations of poor training on security and confidentiality which could affect care [50,51]. It was suggested that investment in training with standardized educational materials was needed at the commencement of employment and on the job [52,53]. In this study, about 25% of HIV-OPC staff were fully trained, but 87% of them appeared to have proper security practices and 66% for the protection of data confidentiality. This may due to health authorities’ enforcement of policies and procedures governing access to and use of personal health data. However, it is still important to acknowledge that those who were trained had better practices, and thus training programs are needed, since some personnel may either be untrained or trained but not fully understand the importance of this matter. Moreover, technical support staff may be required to change personnel behaviors.

Knowledge and perceptions regarding the security and confidentiality of patient information were found to affect the proper practices of HIV-OPC staff, particularly regarding to security measures. Although there were no statistically significant differences for practices of protection of personal health information, the trends appeared to be similar—staff with moderate/good knowledge and perceptions had better practices for safeguarding patient data. Compared with 58% of the general population in a semi-urban setting in another study [54], 88% of HIV-OPC staff had good or moderate knowledge about the confidentiality of HIV-information. However, healthcare providers are expected to have good, rather than moderate, levels of knowledge in this regard. Moreover, different people may have different perceptions of what should be considered sensitive information; thus, it is important to have standards when providing care services to ensure compliance with and responsibility for the administration, monitoring, and evaluation of patient information [55]. To change the perception or attitude of staff members, they must recognize and understand what and why changes are needed. Highlighting the importance of information confidentiality and security measures is essential, as is building relevant technical skills.

The findings of this study reflect the classic theories of associations between knowledge and perception with awareness and behavior. Behavioral theories have also suggested that training is important as it would have positive associations with knowledge, beliefs, perceptions, and behaviors [56, 57]; therefore, arranging continual training on guidelines and regulations related to data security and confidentiality may help improve practices at the healthcare settings, at existing HIV-OPCs or integrated general healthcare hospitals.

Vietnam has regulations for the prevention and control of disclosure of HIV-identifiable information, and penalties for violations of the law [58], but the enforcement and monitoring of the guideline requirements/procedures have not been thorough. Adopting core standards of other countries for data security and confidentiality could be beneficial for HIV-OPC management in Vietnam. For example, stringent core technical standards implemented by the Centers for Disease Control and Prevention [23,26] include the following: (1) Healthcare providers with access to patient-identifiable data should attend data security/confidentiality training annually, and (2) newly hired staff should sign, and all other personnel re-sign, a confidentiality agreement annually. Core standards for data sharing and release include, for example, assessment of risks and benefits of sharing data if the data are to be shared beyond the originally stated purpose. However, a study across several countries concluded that successful development and implementation of guidance requires strong collaboration at local and national levels [12].

### Limitations of the study

Several limitations should be acknowledged in this study. First, we used an observational survey design, and could only assess associations, not causation, between practices and potential factors, such as knowledge and perceptions. Second, several other organizational and structural factors might influence staff practices. As suggested in one guideline, the privacy and security of electronic health information might depend on the following: administrative safeguards, physical safeguards, organizational standards, and policies and procedures [59]. It should also be noted that “professions” was used as a proxy indicator of different types of OPC staffs using or accessing to confidential patient information but it may not reflect the actual respective roles and levels of responsibility among the respondents in handling such information.

## Conclusions

In general, the practices among HIV-OPC staff for securing and protecting patient information were at acceptable levels. Most staff had proper practices for maintaining data security; however, protection of patient confidentiality, particularly for data access, sharing, and transfer, still needed improvement. Most HIV-OPC staff had good or moderate knowledge and positive perceptions towards security and confidentiality issues. Staff with good and moderate knowledge and perception tended to engage in proper practices more often than those needing improvement. Training in data security and confidentiality is an important factor for better practices, as it would also improve knowledge and raise awareness about the need to change the perceptions/attitudes of those needing improvement.

As the operation and management of HIV treatment and care in Vietnam is in transition from separate healthcare clinics (HIV-OPCs) into units integrated into general hospitals/healthcare facilities, the findings of this study suggest issues that might be useful for revising guidelines and regulations by Vietnam health authorities or other countries in similar situations. In planning for development and implementation of the computerized system and case-management procedures during this transition period, one should take into consideration, and have clear strategies to ensure, information confidentiality/safety/security and the benefits for HIV patients. Although there are many advantages to this transition approach, concerns involve the confidentiality of data that might engender patient stigma and discrimination. One of the critical questions of concern in the plan is how to ensure the security and confidentiality of HIV patients’ information when this information will be shared with third parties, especially health insurance officers and other health workers at general hospitals and commune health stations where HIV patients are administered ARV drugs. Secured infrastructure and data access and use measures are a very important and worthwhile investment. The provision of continuous training and active enforcement and monitoring of the practices of healthcare personnel, in either HIV-OPC settings or HIV/AIDS units integrating into general hospitals should improve understanding and acknowledegement of the importance of national policies and guidelines regarding HIV-related patient information.

## Supporting information

S1 Appendix Questionnaire. (DOCX)
