# Supplementary material for: Practices in security and confidentiality of HIV/AIDS patients’ information: A national survey among staff at HIV outpatient clinics in Vietnam
Source: PLoS One. 2017 Nov 14;12(11):e0188160. doi: 10.1371/journal.pone.0188160 (PMC5685590; doi:10.1371/journal.pone.0188160)
Supplement: S1 Appendix — (DOCX) [file pone.0188160.s001.docx]

**QUESTIONNAIRE (ENGLISH)**

**Introduction: survey information and some concepts**

The participation is completely voluntary. This is the study with title “Knowledge, attitude/perception and practice regarding security and confidentiality of HIV-related information among staff at OPC in Vietnam”. The study was approved by the Vietnam Authority of HIV/AIDS Control, Ministry of Health. In order to have mutual understanding of the terms, the operational definitions of the terms are defined as follow:

***Confidentiality*** relates to the right of individuals to protection of their data during storage, transfer, and use, in order to prevent unauthorized disclosure of that information to third parties

***Security*** is a collection of technical approaches that address issues covering physical, electronic, and procedural aspects of protecting information collected as part of the scale-up of health services.

Please answer all questions honestly about your understanding and practices. There is no link to who you are in the questionnaire. The questionnaire is sent out to all OPCs in the country. We hope to assess the situation in Vietnam across country in general, not focusing on any individual location. Your information will help us design the system and strategies for providing proper healthcare services for HIV patient.

**Section 1: Personal Information**

1. *Gender***: Male Female**
2. *Position*: **Doctor Pharmacist Nurse Administration**

**Other, specify**:………………………………………………………

1. *Education*: **Bachelor MD Master Ph.D. Nurse**

**Other, specify**: ……………………………………………………

1. *Do you ever been trained on data security/confidentiality*: **Yes No**
2. *Number of years working in HIV field*: …………………………………………
3. *Number of years working in OPC*: ……………………………………………...

**Section 2: Knowledge**

Please, give your answer for below statements

| **Statement** | **Correct** | **Incorrect** | **Unknown** |
| --- | --- | --- | --- |
| *7. Confidential of patient information is about not disclosing patient’s information to anyone if you have no agreement of patients or law* |  |  |  |
| *8. Authentication sharing of HIV patient information is not one of rules within Vietnamese regulations* |  |  |  |
| *9. Lock cabinet/room/place or set password on computer/apps are ways to ensure data security* |  |  |  |
| *10. There is no Vietnamese regulations about storing HIV patient information* |  |  |  |
| *11. There is no need to concern about storing or using information of HIV patient who was dead.* |  |  |  |
| *12. Doctor and nurse who directly care and treatment HIV patient have right to access patient information* |  |  |  |
| *13. In Vietnam, disclosing HIV status have to be executed in accordance with regulations* |  |  |  |
| *14. Using the antivirus software/encrypting data that is not way to protect patient information* |  |  |  |
| *15. In Vietnam, t**he paper health record of HIV patient need to confidentially stamped on the record* |  |  |  |
| *16. Contracting the guard for 24/24h the OPC is way to ensure security of the patient information* |  |  |  |

**Section 3: Attitude/Perception**

Please, indicate your level of agreement with each of the following statements:

(1: Strongly Agree; 2: Agree; 3: Neutral; 4: Disagree; 5: Strongly disagree)

| **Statement** | **1** | **2** | **3** | **4** | **5** |
| --- | --- | --- | --- | --- | --- |
| **Perceived benefits** | | | | | |
| *17. No matter how well HIV patient information is kept securely, it still won’t help patients facing with the social stigma-related* |  |  |  |  |  |
| *18. Disclosing patients information in accordance with regulations would result in positive effect to both patient and society* |  |  |  |  |  |
| **Perceived threats** | | | | | |
| *19. Setting password on the computer is necessary only on the computer that resides in the public place, and not necessary for the computer in the locked doctor’s room.* |  |  |  |  |  |
| *20. Healthcare providers need to control the patient information closely when using or sharing to others* |  |  |  |  |  |
| *21. Healthcare providers can use their own personal computers to store the patient’s sensitive data* |  |  |  |  |  |
| **Perceived self-efficacy to perform action** | | | | | |
| *22. Health workers don’t have direct responsibility to protect the patient information as it is solely the responsibility of health organization management* |  |  |  |  |  |
| *23. Ensuring security and confidentiality of HIV patient information should be in accordance with the requirements stated in health ethic and law* |  |  |  |  |  |
| *24. In order to prevent risk, HIV status of patient have to be informed to all the health workers in the workplace* |  |  |  |  |  |
| *25. A confidentiality agreement is necessary for all staffs who have access to the data.* |  |  |  |  |  |

**Section 4: Practice**

Please, self-evaluate your practice level with each of the following statements:

**(Always**: more than 80%; **Often**: more than 50%; **Rarely**: less than/equal 50%; **Never**: 0%)

| **Statements** | **Always** | **Often** | **Rarely** | **Never** |
| --- | --- | --- | --- | --- |
| *26. Set and change periodically password for the computer where store the patient information* |  |  |  |  |
| *27. Share patient’s information with other trusted healthcare personnel for consultation purpose* |  |  |  |  |
| *28. Keep patient’s paper records in secured locked cabinet/room at the end of day* |  |  |  |  |
| *29. Enter the patient data on the personal computer* |  |  |  |  |
| *30. Install and use antivirus software on the computer containing sensitive data of HIV patient* |  |  |  |  |
| *31. Send report of the HIV patient information via internet by normal email (not authentication)* |  |  |  |  |
| *32. Ask for consent of HIV patient whether you can share his/her personal information to anyone (e.g., relative or close friend) that he/she allow* |  |  |  |  |

Thank you very much for your cooperation!

**QUESTIONNAIRE (VIETNAMESE)**

**BỘ CÂU HỎI DÀNH CHO NHÂN VIÊN LÀM VIỆC TẠI PHÒNG KHÁM NGOẠI TRÚ HIV**

**Giới thiệu: Thông tin về khảo sát và một số các khái niệm**

Sự tham gia của anh/chị là hoàn toàn tự nguyện. Đây là nghiên cứu với tiêu đề “*Kiến thức, Thái độ, và Thực hành về vấn đề an toàn và bảo mật thông tin liên quan HIV trong nhóm nhân viên làm việc tại phòng khám ngoại trú HIV ở Việt Nam*”. Nghiên cứu đã được sự Cục Phòng, chống HIV/AIDS, Bộ Y tế đồng ý. Để có những hiểu biết về khái niệm, những khái nhiệm đã được định nghĩa như bên dưới đây:

**Bảo mật**: liên quan tới quyền của các cá nhân để bảo vệ dữ liệu của họ trong suốt quá trình lưu trữ, chuyển gửi và sử dụng để ngăn ngừa những tiết lộ trái phép hoặc không chính đáng cho bên thứ ba

**An toàn**: là một tập hợp những kỹ thuật tiếp xử lý các vấn đề bao gồm các khía cạnh như vật lý, điện tử và thủ tục của việc bảo vệ thông tin đã được thu thập như một phần thêm vào của dịch vụ sức khỏe

Xin vui lòng trả lời tất cả các câu hỏi một cách thành thật về những hiểu biết và thực hành của anh/chị. Không có bất cứ thông tin liên kết nào để xác định bạn trong các câu hỏi này. Bộ câu hỏi được gửi đến toàn bộ OPCs trên cả nước. Chúng tôi hi vọng biết được tình hình hiện tại ở Việt Nam nói chung, không tập trung vào bất kỳ khu vực riêng nào. Thông tin của bạn sẽ giúp chúng tôi thiết kế hệ thống và chiến lược cho việc cung cấp những dịch vụ chăm sóc sức khỏe cho bệnh nhân HIV một cách thích hợp hơn.

**Phần 1: Thông tin cá nhân**

1. *Giới tính***: Nam Nữ**
2. Vị trí làm việc: **Bác sĩ Dược sĩ Y tá Hành chính**

**Khác**:………………………………………………………......

1. *Trình độ học vấn*: **Cử nhân Bác sĩ Thạc sĩ Tiến sĩ Y tá**

**Khác**: ……………………………………………………..............

1. *Bạn có từ được tập huấn về án toàn và bảo mật dữ liệu?*  **Có Không**
2. *Số năm kinh nghiệm làm việc lĩnh vực HIV/AIDS*: ……………………….........
3. *Số năm kinh nghiệm làm việc tại phòng khám ngoại trú ARV*: ………………...

**Phần 2: Hiểu biết**

Xin vui lòng đưa ra câu trả lời của anh/chị cho những phát biểu dưới đây

| **Nội dung của phát biểu** | **Chính xác** | **Không chính xác** | **Không biết** |
| --- | --- | --- | --- |
| *7. Bảo mật thông tin của bệnh nhân là không tiết lộ thông tin của bệnh nhân cho bất kỳ ai nếu không có sự đồng ý của họ hay pháp luật* |  |  |  |
| *8. Việc chia sẻ thông tin của bệnh nhân HIV có xác thực đảm bảo không phải là một nguyên tắc trong quy định của Việt Nam* |  |  |  |
| *9. Việc khóa tủ/phòng/nơi làm việc hoặc thiết lập mật khẩu trên máy tính/trên các ứng dụng là cách để đảm bảo an toàn dữ liệu* |  |  |  |
| *10. Ở Việt Nam không có quy định nào về việc lưu trữ thông tin bệnh nhân HIV* |  |  |  |
| *11. Không cần quan tâm về việc lưu trữ, sử dụng thông tin của bệnh nhân HIV đã chết* |  |  |  |
| *12. Bác sĩ, y tá chăm sóc và điều trị trực tiếp cho bệnh nhân có quyền truy cập vào thông tin cá nhân của bệnh nhân* |  |  |  |
| *13. Ở Việt Nam, việc tiết lộ thông tin tình trạng HIV phải được thực hiện theo đúng quy trình* |  |  |  |
| *14. Sử dụng phần mềm diệt virus/mã hóa dữ liệu không phải là cách bảo vệ thông tin bệnh nhân* |  |  |  |
| *15. Ở Việt Nam, hồ sơ bệnh án giấy cần phải đóng dấu “Mật” vào hồ sơ* |  |  |  |
| *16. Hợp đồng với người bảo vệ 24/24 giờ cũng là cách để đảm bảo an toàn thông tin cho bệnh nhân* |  |  |  |

**Phần 3: Thái độ**

Xin vui lòng chỉ ra mức độ đồng ý của anh/chị cho mỗi phát biểu dưới đây:

(**1**: Rất Đồng ý; **2**: Đồng ý; **3**: Trung lập; **4**: Không Đồng ý; **5**: Rất không Đồng ý)

| **Nội dung phát biểu** | **1** | **2** | **3** | **4** | **5** |
| --- | --- | --- | --- | --- | --- |
| **Nhận thức về lợi ích** | | | | | |
| *17. Dù thông tin bệnh nhân có được giữ bí mật thế nào đi nữa, nó cũng không giúp bệnh nhân tránh được phân biệt đối xử xã hội* |  |  |  |  |  |
| *18. Việc tiết lộ thông tin bệnh nhân HIV phù hợp với các quy định sẽ có những ảnh hưởng tích cực tới bệnh nhân và xã hội* |  |  |  |  |  |
| **Nhận thức các nguy cơ** | | | | | |
| *19. Thiết lập mật khẩu trên máy tính chỉ cần thiết trên máy tính đặt ở những khu vực chung và không cần thiết với máy tính đặt ở trong phòng của bác sĩ đã được khóa* |  |  |  |  |  |
| *20. Những nhân viên chăm sóc sức khỏe cần kiểm soát chặt chẽ thông tin của bệnh nhân khi sử dụng và chia sẻ với người khác* |  |  |  |  |  |
| *21. Nhân viên chăm sóc sức khỏe có thể dùng máy tính cá nhân của họ để lưu trữ những dữ liệu nhạy cảm của bệnh nhân.* |  |  |  |  |  |
| **Nhận thức sự tự hiệu quả để thực hành** | | | | | |
| *22. Nhân viên y tế không có trách nhiệm trực tiếp để bảo vệ thông tin bệnh nhân vì nó là trách nhiệm của nhà quản lý tổ chức y tế* |  |  |  |  |  |
| *23. Bảo đảm án toàn và bảo mật thông tin của bệnh nhân HIV là phù hợp với những yêu cầu đã được nêu trong y đức và luật pháp* |  |  |  |  |  |
| *24. Để ngăn ngừa những nguy cơ, tình trạng HIV của bệnh nhân phải được thông báo cho tất cả các nhân viên y tế ở nơi làm việc* |  |  |  |  |  |
| *25. Một bản cảm kết đảm bảo Bí mật là cần thiết cho tất cả những nhân viên - người tiếp cận được với dữ liệu* |  |  |  |  |  |

**Phần 4: Thực hành**

Xin vui lòng tự đánh giá mức độ thực hành của anh/chị cho mỗi phát biểu dưới đây:

**(Luôn luôn**: hơn 80%; **Thường xuyên**: hơn 50%; **Ít khi**: nhỏ hơn/bằng 50%; **Không bao giờ**: 0%)

| **Nội dung** | **Luôn luôn** | **Thường xuyên** | **Ít khi** | **Không bao giờ** |
| --- | --- | --- | --- | --- |
| *26. Thiết lập và thay đổi mật khẩu định kỳ cho máy tính chứa thông tin bệnh nhân* |  |  |  |  |
| *27. Chia sẻ thông tin của bệnh nhân với những nhân viên y tế tin tưởng khác để tham khảo ý kiến* |  |  |  |  |
| *28. Giữ bệnh án của bệnh nhân một trong tủ/phòng được khóa an toàn khi hết ngày làm việc* |  |  |  |  |
| *29. Nhập dữ liệu bệnh nhân trên máy tính cá nhân* |  |  |  |  |
| *30. Cài đặt và sử dụng phần mềm diệt virus trên máy tính có chứa dữ liệu nhạy cảm của bệnh nhân* |  |  |  |  |
| *31. Gửi báo cáo thông tin bệnh nhân qua thư điện tử thường (không đảm bảo)* |  |  |  |  |
| *32. Yêu cầu sự đồng ý của bệnh nhân cho dù bạn có thể chia sẻ thông tin cá nhân của họ cho bất cứ ai (ví dụ: người thân hay bạn thân)* |  |  |  |  |

Xin cảm ơn sự hợp tác của anh/chị!
